# Could *Miscanthus* replace maize as the preferred substrate for anaerobic digestion in the United Kingdom? Future breeding strategies

**DOI:** 10.1111/gcbb.12419

**Published:** 2017-01-21

**Authors:** Sarah J. Purdy, Anne L. Maddison, Christopher P. Nunn, Ana Winters, Emma Timms‐Taravella, Charlotte M. Jones, John C. Clifton‐Brown, Iain S. Donnison, Joe A. Gallagher

**Affiliations:** ^1^ Institute of Biological Environmental and Rural SciencesAberystwyth UniversityPlas GogerddanCeredigionSY23 3EBUK

**Keywords:** anaerobic digestion, bioenergy, carbohydrates, *Miscanthus*, miscanthus breeding, starch, sugar

## Abstract

Fodder maize is the most commonly used crop for biogas production owing to its high yields, high concentrations of starch and good digestibility. However, environmental concerns and possible future conflict with land for food production may limit its long‐term use. The bioenergy grass, *Miscanthus*, is a high‐yielding perennial that can grow on marginal land and, with ‘greener’ environmental credentials, may offer an alternative. To compete with maize, the concentration of non‐structural carbohydrates (NSC) and digestibility may need to be improved. Non‐structural carbohydrates were quantified in 38 diverse genotypes of *Miscanthus* in green‐cut biomass in July and October. The aim was to determine whether NSC abundance could be a target for breeding programmes or whether genotypes already exist that could rival maize for use in anaerobic digestion systems. The saccharification potential and measures of N P and K were also studied. The highest concentrations of NSC were in July, reaching a maximum of 20% DW. However, the maximum yield was in October with 300–400 g NSC plant^−1^ owing to higher biomass. The digestibility of the cell wall was higher in July than in October, but the increase in biomass meant yields of digestible sugars were still higher in October. Nutrient concentrations were at least twofold higher in July compared to November, and the abundance of potassium showed the greatest degree of variation between genotypes. The projected maximum yield of NSC was 1.3 t ha^−1^ with significant variation to target for breeding. Starch accumulated in the highest concentrations and continued to increase into autumn in some genotypes. Therefore, starch, rather than sugars, would be a better target for breeding improvement. If harvest date was brought forward to autumn, nutrient losses in non‐flowering genotypes would be comparable to an early spring harvest.

## Introduction

*Miscanthus* is a bioenergy grass predominantly used for heat and power (Jensen *et al*., 2016). It is a perennial species that produces high annual yields and requires very low chemical inputs (Lewandowski *et al*., 2000). There are two main subspecies of *Miscanthus: M. sinensis* and *M. sacchariflorus*. The commercially grown genotype, *M. x giganteus,* is a hybrid between the two species. *M. x giganteus* genotypes are the progeny of a tetraploid, Japanese *M. sacchariflorus,* and a diploid, Japanese *M. sinensis*; this combination has proved to produce high‐yielding plants from multiple, independent crossing events with different parents (Wang *et al*., 2008a; Jezowski *et al*., 2011; Purdy *et al*., 2013). As a member of the subtropical Poaceae, *Miscanthus* is related to two other major food and bioenergy crops: maize and sugarcane (Hodkinson *et al*., 2002).

The soluble sugar content of actively growing *M. x giganteus* clones has been reported to be approximately 6% DW (Purdy *et al*., 2013; de Souza *et al*., 2013). In a study of four genotypes of *Miscanthus* representing both species and an *M. x giganteus*, peak‐soluble sugar contents were 6–8% (Purdy *et al*., 2014). This is comparable to sugarcane progenitors (Wang *et al*., 2008b; Lingle *et al*., 2009; de Souza *et al*., 2013), which has led to the proposition that *Miscanthus* could be bred to produce a temperate sugarcane (de Souza *et al*., 2013). However, unlike sugarcane, *Miscanthus* also accumulates starch to concentrations ranging between 2% and 7% DW in the shoots depending upon genotype (de Souza *et al*., 2013; Purdy *et al*., 2014). This then raises the possibility that instead of breeding for soluble sugars, with potential problems of feedback inhibition of photosynthesis, the focus could switch to increasing starch content. Elevated levels of nonstructural carbohydrates (NSC) would broaden the potential uses of *Miscanthus* from being burnt for fuel, to being a feedstock for anaerobic digestion (AD). Anaerobic digestion is the decomposition of organic matter in an anaerobic environment to produce biogas that is usually around 60% methane and 40% carbon dioxide (DECC & DEFRA, 2011, Whittaker *et al*., 2016). Biogas can be produced from a variety of organic wastes, animal manures or energy crops (Amon *et al*., 2007). Forage maize is the most commonly used crop for AD (Mayer *et al*., 2014a) and plant breeders have bred tailored varieties specifically for AD. New varieties of forage maize for AD are early maturing, have a high dry matter (DM) yield (>30%), high starch yield of ~6 t ha^−1^, high digestibility and high metabolizable energy (ME; BSPB, 2016). The use of forage maize for AD has increased rapidly across Europe, particularly in Germany, but this has raised concerns about the negative effects on soil and waterway health and competition between land for fuel and food (Weiland, 2006; Klimiuk *et al*., 2010; Mayer *et al*., 2014b; Kiesel & Lewandowski, 2016). In a study into soil health and land use in south‐west England, soils under maize and potatoes had the most degraded soils, with 75% of sites exhibiting erosion (Palmer & Smith, 2013). This is linked to increased overland flow of water across fields and into waterways which, in turn, causes water pollution and localized flooding (Palmer & Smith, 2013). *Miscanthus* has been identified as being the most promising alternative to maize for biogas yield compared to 13 other possible AD substrates (Mayer *et al*., 2014b). The higher yields of *Miscanthus* in continental Europe mean that *Miscanthus* can already compete with the biogas yields of maize, with methane yields of 6153 m^3^ ha^−1^ and 6008 m^3^ ha^−1^ for Miscanthus and maize, respectively (Kiesel & Lewandowski, 2016). In a recent study, forage maize had sugar and starch contents of ~8% and ~18%, whereas *M. x giganteus* has sugar and starch contents of ~5% and 4%, respectively, and a BMP of less than half that of maize (Whittaker *et al*., 2016). The study concluded that to compete with maize for AD, *Miscanthus* yields would have to be increased from ~14 to 19–26.5 t ha^−1^ (Whittaker *et al*., 2016), but another possible scenario would be to also increase the concentration of starch and/or soluble sugars in *Miscanthus* through breeding.

A major difference between Miscanthus and maize is the concentration of starch and cellulose. Miscanthus predominantly accumulates cellulose (~35% DW) rather than starch (~4% DW) whereas maize accumulates a higher proportion of starch (~18% DW) compared to cellulose (13% DW; Whittaker *et al*., 2016). Although starch and cellulose are both polymers of glucose, starch is the preferred substrate for AD because it is easier to breakdown (Montgomery & Bochmann, 2014). The limiting factor for cellulose is its physical and chemical association with lignin which is not digestible in anaerobic conditions and impedes the breakdown of the cell wall polysaccharides (Weng *et al*., 2008). When using lignocellulosic materials, such as straw, in an AD system, the high levels of recalcitrance mean that only 40–50% of the feedstock is converted to biogas and the rest is unused (Ahring *et al*., 2015). Conversely, a reactor fed on late‐harvested maize achieved 84% of the theoretical biogas potential (Bruni *et al*., 2010). Therefore, a higher abundance of starch, rather than lignocellulose, and high digestibility are desirable for maximizing biogas outputs.

At present, the concentration of starch in *M. x giganteus* at peak yield in west Wales is approximately 5% DW. With peak autumn yields of 16 t ha^−1^, this equates to approximately 0.8 t ha^−1^ which is 7.5‐fold less than the yield of starch from forage maize. However, most studies of NSC concentrations in *Miscanthus* have focussed on a limited range of genotypes (mainly *M. x giganteus*), and the amount of natural diversity in NSC content available in other genotypes is unclear. To address the possibility of identifying genotypes better suited to AD or exploiting natural diversity to breed new varieties, we sought to quantify soluble sugars and starch in a diverse range of germplasm.

*Miscanthus* is usually harvested at the very end of winter (January–March in the northern hemisphere) when it is fully senesced. By this time, carbohydrates and mineral nutrients accumulated over the growing season have been remobilized to the underground rhizome, and the stems are dry (Robson *et al*., 2012; Purdy *et al*., 2014). Therefore, if carbohydrates were to be captured, the crop would have to be harvested green. This then presents a number of dilemmas; a major attribute of *Miscanthus* is its low (usually nil) fertilizer demands owing to its efficient recycling system. If the stems are harvested before nutrient remobilization has taken place, the essential nutrient elements such as nitrogen (N), phosphate (P) and potassium (K) may also be removed and will require replacement with fertilizers to restore productivity. If *Miscanthus* is harvested green, it also needs to be stored to prevent nutrient losses necessary for the AD process. Studies have now shown that *Miscanthus* can ensile well‐producing good‐quality silage but quality depends upon harvest date (Klimiuk *et al*., 2010; Whittaker *et al*., 2016). It has previously been shown that in four genotypes of *Miscanthus*, rhizome NSC had been replenished to winter levels by September suggesting that harvesting after this month may not leave the rhizome depleted of carbohydrates (Purdy *et al*., 2014). To address the implications of early harvesting on elemental nutrients, we quantified N, P and K from samples harvested in July, November–December and January over 2 years.

If harvest date was to be shifted to capture NSC for AD, the biomass must be readily digestible to maximize yields of biogas as biomethane production is influenced by lignocellulosic digestibility (Hendriks & Zeeman, 2009). However, as with all lignocellulosic fermentation, the challenge is releasing the carbohydrates from their recalcitrant form to increase availability to the microbial population. As the plant matures, the composition of the cell wall also changes (da Costa *et al*., 2014). In *Miscanthus*, lignin increases during maturity and this is negatively correlated with biogas production as it blocks access to the cellulose chains by cellulases (Klimiuk *et al*., 2010; Ngoc Huyen *et al*., 2010). To assess whether there would be an advantage of shifting the harvest date on the saccharification potential, we analysed this parameter at the two time points.

The questions that we addressed in this study were as follows:

1. What is the range in variation for NSC abundance across diverse genotypes?
2. If harvest date was to be moved to capture maximum NSC, when should this occur?
3. What are the implications for saccharification potential and N P and K removal of harvesting at earlier time points?

## Materials and methods

Nonstructural carbohydrate composition and saccharification potential.

### Plant material

Details of all the individual genotypes used in these experiments are shown in Table 1.

**Table 1:** Details of the *Miscanthus* genotypes used in the four experimental procedures

	Species	Ploidy	Country of origin (where known)	Field trials and genotypes previously cited in
**Name**	**Experiment: nonstructural carbohydrate and saccharification**			
Sin 1	*sinensis*	2		Allison *et al*. (2011), da Costa *et al*. (2014), Jensen *et al*. (2011), Jensen *et al*. (2013), Robson *et al*., (2013a,b)
Sin 2	*sinensis*	2		
Sin 3	*sinensis*	2		
Sin 4	*sinensis*	2		
Sin 5	*sinensis*	2	Japan	
Sin 6	*sinensis*	2	Japan	
Sin 7	*sinensis*	2	South Korea	
Sin 8	*sinensis*	2	South Korea	
Sin 9	*sinensis*	2	South Korea	
Sin 10	*sinensis*	2	South Korea	
Hyb 1	*sinensis x sacchariflorus*	3		
Hyb 2	*sinensis x sacchariflorus*	3		
Hyb 3	*sinensis x sacchariflorus*	3		
Hyb 4	*sinensis x sacchariflorus*	4		
Sac 2	*Sacchariflorus var lutarioriparius*	2	China	
Sac 3	*sacchariflorus*	2	China	
Sac 4	*Sacchariflorus var robustus*	2	China	
Sac 5	*sacchariflorus*	4	Japan	
Goliath	*sinensis*	3	Japan	
Hyb 5–23	*sinensis x sacchariflorus var robustus*	2		
**Name**	**Experiment: N, P and K analysis**			
Sac 5	*sacchariflorus*	4	Japan	Davey *et al*., (2016), Purdy *et al*. (2013, 2014, 2015)
Gig‐311	*sinensis x sacchariflorus*	3	Japan	
EMI‐11	*sinensis*	2	Japan	
Goliath	*sinensis*	3	Japan	
**Name or number in each species**	**Experiment: modelling crop yield as % of final harvest mass**			
3	*sacchariflorus*	4		
1	*sacchariflorus robustus*	2	China	
5	*sinensis x sacchariflorus*	2		
Goliath	*sinensis*	3	Japan	
3	*sinensis*	Unknown		

### Mixed population

In 2004 at Aberystwyth, west Wales, United Kingdom, a total of 244 *Miscanthus* genotypes were collected and planted as spaced plants as previously described (Allison *et al*., 2011; Jensen *et al*., 2011; Robson *et al*., 2012). From this population, 18 genotypes representing 10 *M. sinensis*, four *M. sinensis* x *M. sacchariflorus* hybrids and four *M. sacchariflorus* were selected. Three biological replicates per genotype were harvested from blocks 1, 2 and 3 of the trial. The numbering system for the four *M. sacchariflorus* genotypes is Sac 2‐Sac 5 with no Sac 1. This is because ‘Sac 5’ has been previously included in other studies (Purdy *et al*., 2013, 2014, 2015), and so to maintain consistency, genotypes were numbered to include Sac 5 which meant omission of a ‘Sac 1’.

### Mapping family

A total of 102 genotypes from a paired cross between a diploid *M. sinensis,* similar to Sin 5, and diploid *M. sacchariflorus robustus* genetically indistinguishable from Sac 4 were sown from seed in trays in a glasshouse in 2009. In 2010, individual plants were split to form three replicates of each genotype and then planted out into the field in a spaced‐plant randomized block design comprising three replicate blocks. The field site is located 300 m to the south from the mixed population (described above), and therefore stone content and soil types are as described previously (Allison *et al*., 2011).

### Destructive harvests

A single stem that was representative of canopy height was selected from each plant, cut at a height of 10 cm from the base and then flash‐frozen before freeze‐drying. As NSC show diurnal fluctuations in *Miscanthus* (Purdy *et al*., 2013), the two sets of plants were harvested on different days in July and October so that each harvest could be completed within a 2‐h window at the same time of day (Zt 8–10 of a 16‐h photoperiod).

Annual yield harvest: The mixed population and mapping family were destructively harvested for yield in March 2014 (following the 2013 growing season). Biomass was dried to a constant weight, and then the average DW weight per plant (kg) was calculated.

### Non‐structural carbohydrates (NSC) compositional analyses

Soluble sugars and starch were analysed as previously described (Purdy *et al*., 2014, 2015). Soluble sugar extraction: Approximately 20 mg DW (actual weight recorded) of each cryomilled (6870 Freezer Mill, Spex, Sampleprep, Stanmore, UK) plant tissue sample was weighed into 2‐mL screwcap microcentrifuge tubes. Sugars were extracted four times with 1 mL of 80% (v/v) ethanol and the resulting supernatants pooled; two extractions were at 80 °C for 20 min and 10 min, respectively, and the remaining two at room temperature. A 0.5‐mL aliquot of soluble sugar extract and the remaining pellet containing the insoluble fraction (including starch) were dried‐down in a centrifugal evaporator (Jouan RC 1022, Saint Nazaire, France) until all the solvent had evaporated. The dried‐down residue from the soluble fraction was then resuspended in 0.5 mL of distilled water. Samples were stored at −20 °C for analysis.

Soluble sugar analysis: Soluble sugars of samples extracted in the previous step were quantified enzymatically by the stepwise addition of hexokinase, phosphoglucose isomerase and invertase (Jones *et al*., 1977). Samples were quantified photometrically (Ultraspec 4000; Pharmacia Biotech, Sweden) by measuring the change in wavelength at 340 nm for 20 min after the addition of each enzyme. Sucrose, glucose and fructose were then quantified from standard curves included on each 96‐well plate. Soluble sugar data shown in this paper are the sum of these three sugars.

Starch quantification: Starch was quantified using a modified Megazyme protocol (Megazyme Total Starch Assay Procedure, AOAC method 996.11, Megazyme International, Wicklow, Ireland). Briefly, the dried pellet was resuspended in 0.4 mL of 0.2 m KOH, vortexed vigorously and heated to 90 °C in a water bath for 15 min to facilitate gelatinization of the starch. A total of 1.28 mL of 0.15 m NaOAc (pH 3.8) was added to each tube (to neutralize the sample) before the addition of 20 μL *α*‐amylase and 20 μL amyloglucosidase (Megazyme International). After incubation at 50 °C for 30 min and centrifugation for 5 min, a 0.02‐mL aliquot was combined with 0.6 mL of GOPOD reagent (Megazyme). A total of 0.2 mL of this reaction was assayed photometrically (Ultraspec 4000; Pharmacia Biotech, Uppsala, Sweden) on a 96‐well microplate at 510 nm against a water‐only blank. Starch was quantified from known standard curves on the same plate. Each sample and standard was tested in duplicate. Each plate contained a *Miscanthus* control sample of known concentration for both soluble sugars and starch analysis.

### Total cell wall sugars

Approximately 60 mg DW (actual weight recorded) of plant cell wall material was purified by sequential ethanol extractions to remove soluble sugars, followed by starch digestion. 1.5 mL of chloroform/methanol (1 : 1 v : v) was then added to the pellet, vortexed and centrifuged. The supernatant was discarded, the pellet was washed with distilled water, vortexed and centrifuged, the supernatant discarded, and this step was repeated twice more. The purified cell wall‐enriched fractions were hydrolysed with 0.6 mL of 72% H_2_SO_4_, vortexed and left to incubate whilst shaking at 200 rpm for 1 h at 30 °C. After incubation, samples were diluted with 16.8 mL of deionized H_2_O. Tubes were then capped and autoclaved at 121 °C for 1 h. Once cooled, an aliquot of 0.65 mL was neutralized with 30 mg CaCO_3_ and centrifuged to pellet the CaCO_3,_ and the supernatant was removed to a fresh tube. Glucose, xylose and arabinose content quantified on a Jasco HPLC system (Jasco Ltd, Great Dunmow, Essex, UK). Samples were prepared by combing 250 μL of extract with 750 μL of 5 mm H_2_SO_4_, containing 5 mm crotonic acid as an internal standard. Sugars were separated on a Rezex ROA‐Organic Acid (150 × 7.8 mm) column with a mobile phase of 5 μm H_2_SO_4_ at 0.6 mL min^−1^. Quantification was based on standard curves prepared using sugar standards.

### Saccharification potential

Saccharification potential was estimated by measuring enzymatic digestibility of cell wall polysaccharides from cell wall‐enriched fractions. Prior to enzymatic hydrolysis, samples were subjected to a mild acid pretreatment. Approximately 50 mg (actual weight recorded) of cell wall‐enriched fractions was suspended in 1.6 mL of 1.5% sulphuric acid (w/w) in a screwcap microfuge tube and subsequently autoclaved at 121 °C for 20 min. Pretreated samples were neutralized by the addition of 28 mg of calcium carbonate. An enzyme cocktail, comprised of Accellerase 1500 and Accellerase XY (kindly supplied by DuPont, USA) and Depol 740 L (a ferulic acid esterase; kindly supplied by Biocatalysts Ltd., Cardiff, UK), was made up in 0.1‐m sodium citrate buffer pH 4.8 at the manufacturers’ recommended application rates. Enzymatic hydrolysis was carried out by the addition of 300 *μ*L of the enzyme cocktail to each tube with incubation for 72 h at 50 °C. After this period, enzymes were inactivated by incubation for 10 min at 80 °C. Enzyme digests were analysed for glucose, xylose and arabinose on the Jasco HPLC system as described above with the exception that samples were prepared by adding a volume of 50 μL of sample to 950 μL of a solution of 5 mm H_2_SO_4_ with 5 mm crotonic acid. The extent of hydrolysis was estimated as a percentage of the total cell wall sugar content.

### Quantification of N, P and K

#### Field trial

The four genotypes and trial site used for the quantification of N, P and K are as previously described (Purdy *et al*., 2014; Table 1). In May 2009, as part of the BSBEC‐BioMASS project (http://www.bsbec-biomass.org.uk/), a dedicated trial was established at the Institute of Biological Environmental and Rural Sciences (IBERS), Aberystwyth, west Wales (52.4139’ N, −4.014’ W). The trial was a randomized block design consisting of four blocks, each block containing four plots, one for each *Miscanthus* genotype described in Table 1. Each plot contained 121 plants with areas designated to nondestructive measurements, annual yield harvest and destructive harvests. Plants were grown from rhizome pieces and cut from mature stands in modules before planting at a density of two plants per m^2^. Surrounding each plot was a row of guard plants of the same genotype. The soil type at Aberystwyth is classified as a silty clay loam.

#### Destructive harvests

Whole plants were harvested in July 2011, November 2011, January 2012, July 2012, December 2012 and January 2013 as follows: plants within the designated destructive harvest area in each plot were assigned a number. Harvest sequence was then determined using a random number generator thereby assigning a particular individual to a specific harvest date. At each harvest, a single plant per plot was harvested (*n* = 4). The total above‐ground biomass was then harvested at 10 cm, the material was chipped, and a subsample was taken, flash‐frozen and stored on dry ice until freeze‐drying.

#### N, P and K analyses

Mineral elements were analysed by an in‐house analytical chemistry service. All samples were harvested and milled as described above. Nitrogen (N) was analysed by a rapid combustion method using a LECO FP‐428 analyser (LECO Corp., St. Joseph, MI, USA). For the determination of potassium (K) and Phosphorous (P), 1 g of sample was weighed into 100‐mL Kjeldahl tubes, and 15 mL aqua regia (780 mL HCl; 500 mL HNO_3_; 720 mL H_2_O) was added and allowed to soak overnight. Samples were digested on a heating block at 120 °C for 3 h, allowed to cool and then quantitatively transferred to 50‐mL volumetric flasks. The solutions were filtered through Whatman No 1 filter paper and then analysed using a Varian Liberty ICP‐AES (Agilent Technologies, Santa Clara, CA, USA).

### Yield modelling

#### Field trial

A randomized block design field trial was planted at Aberystwyth in May 2012. Each of the replicate blocks contained 15 plots measuring 5 m by 5 m and containing 49 plants, making a planting density of approximately 2 m^−2^. The selected germplasm was a mix of *M. sacchariflorus*,* M. sinensis* and interspecies hybrids of *M. sacchariflorus* with *M. sacchariflorus var robustus* and *M. sinensis* (Table 1). The plots were planted as plug plants, propagated either by *in vitro* cloning or from seedlings.

#### Harvesting method

In 2014 and 2015, at monthly intervals, stems were harvested from each plot to measure the changing stem mass. Two stems were selected at random from four plants in the inner border of each plot. To prevent damage to the plants, the row sampled rotated, with each plant only being sampled twice in a growing season. A final comparison sample was also taken directly prior to the yield harvest in spring. The annual yield harvest was taken in early spring in 2014 and 2015. Total biomass was harvested from nine plants per plot. Samples were weighed for fresh weight then oven dried and weighed again for dry weight and moisture content.

#### Modelling

To model yields through the growing season, the ratio of the sample dry weights to the harvest sample dry weight was calculated for every plot at each time point. These ratios were fitted to a curve using a loess smooth in the r package ggplot2 (Wickham, 2009). Yields were modelled by species, to find the rate of growth, peak yield date and rate of change in biomass from emergence to harvest.

### Statistical analyses

Differences between genotypes and harvest dates for NSC, structural carbohydrates, saccharification potential and NPK were determined from anovas using genotype and date as treatment factors (*P* =≤ 0.05). All analyses were performed using genstat (13th Edition) (VSN International, Hemel Hempstead, UK).

## Results

**Figure 1:**
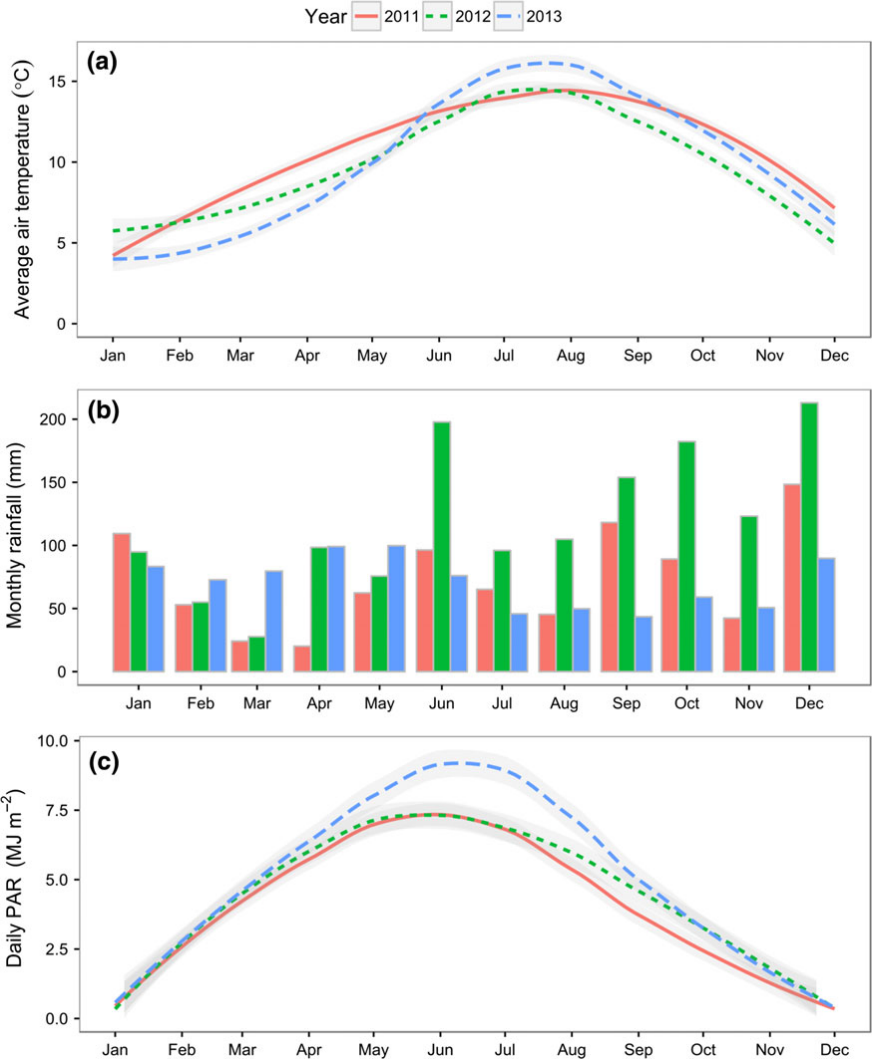
Climatic conditions at Aberystwyth for the years 2011, 2012 and 2013. (a) monthly average air temperature, (b) monthly rainfall and (c) average daily PAR.

The average daily temperature for July in 2013 was 17 °C, whereas in 2011 and 2012 July was cooler at 14 °C (Fig. 1). In October, the temperatures in 2011 and 2013 were similar, 12 °C, but 2012 was 2 degrees cooler (10 °C). In 2013, the average daily rainfall was lower than 2011 and 2012 at 1.5 and 1.9 mm in July and October, respectively. Rainfall in 2012 was unusually high, exceeding 150 mm in June, September, October and December. The average daily PAR in 2013 was 9.9 and 2.5 MJ m^−2^ in July and October, which was considerably higher than the summers of 2011 and 2012 which both averaged 7 MJ m^−2^ in July. PAR was similar in October in 2013 and 2012 but lower in 2011. Therefore, the summer of 2013 was brighter, warmer and drier than the two previous years.

### Quantification of non‐structural carbohydrates

**Figure 2:**
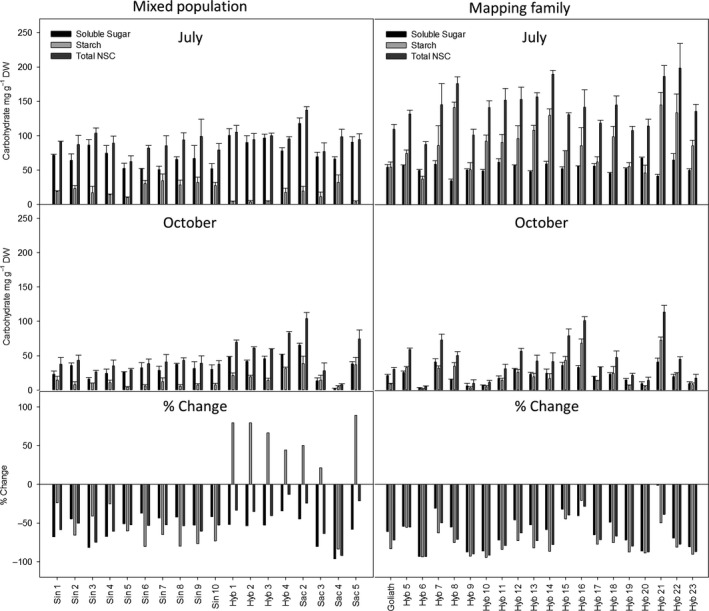
The concentration of non‐structural carbohydrates in a mixed population and a mapping family of *Miscanthus* in July and October and the % change between the two dates. N = 3 ± SE.

The concentration of soluble sugars and total non‐structural carbohydrates (NSC) was significantly higher in both field trials in July compared to November (Date, *P* =< 0.001; Fig. 2 and Table 2). However, in the four hybrid genotypes of the mixed population and three of the four *M. sacchariflorus* genotypes, the concentration of starch increased between July and October. Therefore, whilst differences in date for this set of plants were not significant (*P* = 0.062), a significant interaction between genotype and date was observed (geno × date *P* =< 0.001; Table 2). This trend was not observed in the mapping family. In the mixed population, the majority of NSC was in the form of soluble sugars at both time points. The exceptions to this were the *M. sacchariflorus* genotypes numbers Sac 3, 4 and 5 in October in which the abundance of sugars and starch was similar. In contrast to this, the majority of NSC in the mapping family was in the form of starch in July whereas in October soluble sugars tended to be slightly higher or similar to starch. The exceptions to this were Hyb 8, 16 and 21 in which starch remained the more predominant carbohydrate into autumn (Fig. 2). The maximum concentration of soluble sugar in July was 120 mg g DW^−1^ in Sac 2 of the mixed population and the lowest at 30 mg g DW was from Hyb 8 of the mapping family. Interestingly, the second highest maximum concentration of starch was also observed in Hyb 8 suggesting that there may be a trade‐off between soluble sugars and starch. The maximum concentration of NSC was in the mapping family in July at 200 mg g DW from Hyb 22, and this genotype also retained the highest concentration in October. The lowest concentration of NSC in July was in Sin 5 with 60 mg g DW and in October it was in Sac 4 with 70 mg g DW^−1^ (Fig. 2).

**Table 2:** Statistical analyses of non‐structural carbohydrates (NSC). The effect of harvest date and genotype on NSC. Tests are a two‐way anova with date and genotype as factors. *P* = ≤ 0.05

		*F* pr	
	Soluble sugar	Starch	Total NSC
Mixed population			
Genotype	<0.01	0.012	<0.01
Date	<0.01	0.062	<0.01
Geno × Date	0.001	<0.01	0.02
Mapping family			
Genotype	<0.01	<0.01	<0.01
Date	<0.01	<0.01	<0.01
Geno × Date	<0.01	<0.01	<0.01

### Biomass yield

**Figure 3:**
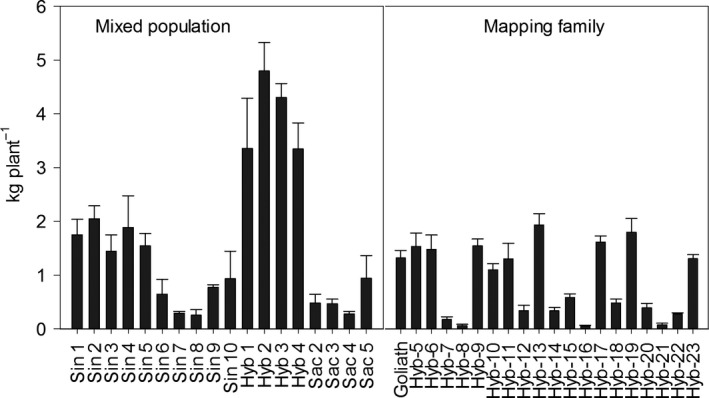
Final yield (kg plant^−1^) for a mixed population and mapping family harvested in spring 2014 following the 2013 growing season. N = 3 ± SE.

The highest yielding plants in spring 2014 (following the 2013 growing season when plants were sampled for NSC in July and October) were the four hybrid genotypes of the mixed population at 3–5 kg DW plant^−1^ (Fig. 3). The highest yielding hybrids of the mapping family were similar in final yield to Sin 1–5 of the mixed population. The lowest yielding plant was Hyb 21 at 0.07 kg (70 g) DW plant^−1^. The *M. sacchariflorus* genotypes were also generally low yielding, especially Sac 2–4 (Fig. 3).

**Figure 4:**
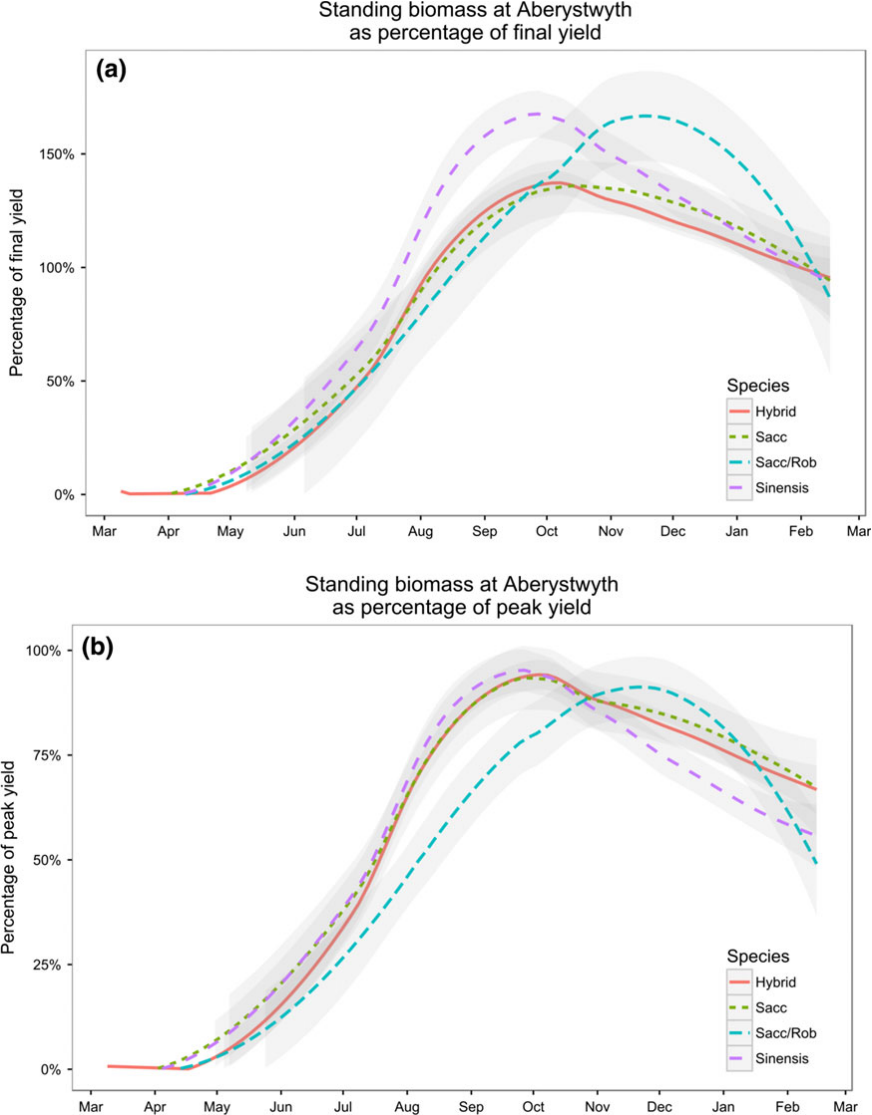
Modelled prediction of biomass as a % of final yield (4a) and (4b) modelled prediction of biomass as a % of peak yield. Data are the mean of 5 × hybrids, 3 × *M. sacchariflorus* (Sacc), 1 × *M*. *sacchariflorus var robustus* (Sacc/Rob) and 3 × *M. sinensis*.

The samples used for the analysis of carbohydrates were taken from single stems harvested in July and October 2013. To project the yields of total carbohydrate in July and October, sequential harvests were taken from a separate field site over a two‐year period (Table 1). The genotypes used were *M. sinensis*,* M. sacchariflorus*,* M. sinensis* × *M. sacchariflorus* hybrids and an *M. sacchariflorus var robustus* which were representative of those in the mixed population and mapping family. At the end of each growing season (January–March), an area of each plot was harvested to give a yield for each genotype in t ha^−1^. The weight of each individual plant at each time point was then modelled as a % of the final harvest mass (Fig. 4a). Although autumn yields have been reported or calculated as a % of final yield in previous publications (Clifton‐Brown *et al*., 1998; Whittaker *et al*., 2016), July yields have not. The mean value of the four genotypes in July was 50% of the final harvest mass which was, on average, 30% of peak, autumn biomass (Fig. 4b). In October, yields were projected to be an average of 40% higher than harvest weight (Fig. 4a). This finding is in close agreement with Kiesel & Lewandowski (2016) who observed that harvested biomass was 39% higher in October compared to February in *M. x giganteus* in Germany.

### Projected NSC yields

**Figure 5:**
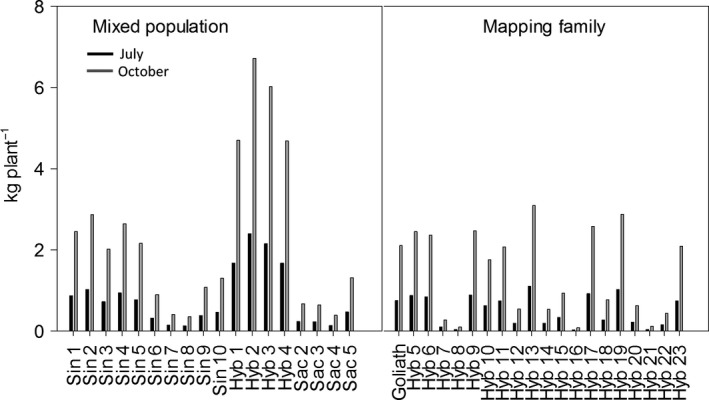
Predicted mass of plants in July and October. Values are based on plants weighting 50% of final harvest mass in July and +40% of harvest mass in October.

**Figure 6:**
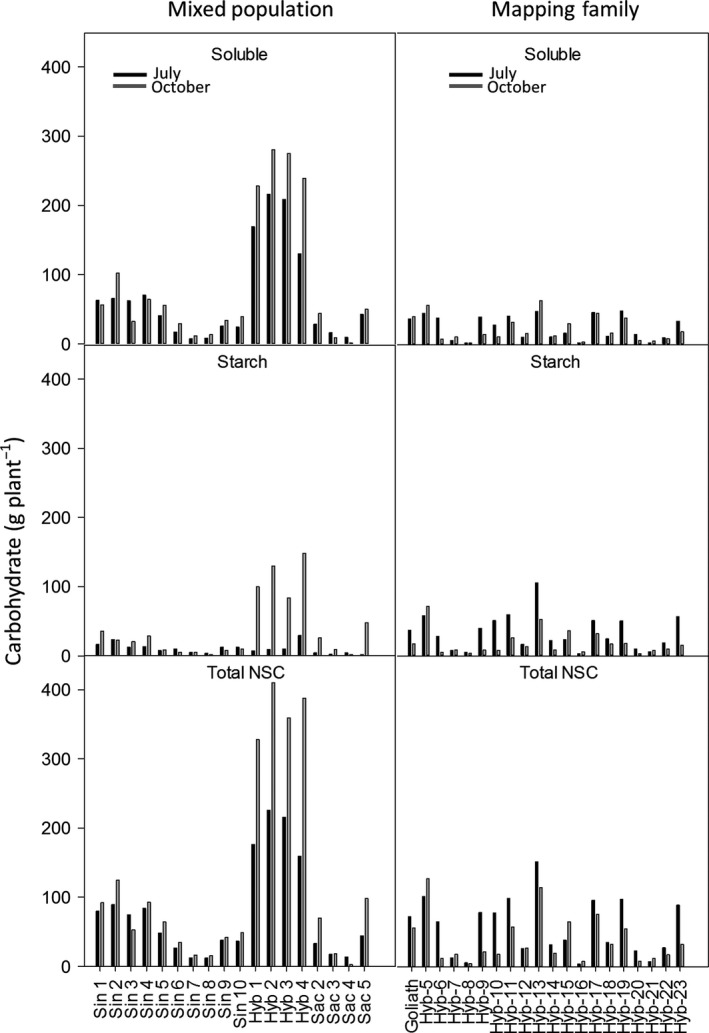
Predicted mass of nonstructural carbohydrates per plant in July and October in a mixed population and hybrid mapping family. Values are based on plants weighting 50% of final harvest mass in July and +40% of harvest mass in October (Fig. 2).

Based on the modelled values and the final yield harvest the following spring (Figs 3 and 4), the mass of plants at the two time points was calculated (Fig. 5) and then used to calculate the DW g of carbohydrate plant^−1^ (Fig. 6). The plants that yielded the highest soluble sugars and starch were the four hybrid genotypes (Hyb 1–4) of the mixed population. The maximum yield of sugar was 280 g plant^−1^ from Hyb 2, and the highest yield of starch was from Hyb 4 at 148 g plant^−1^, both in October. In the mixed population, starch contents increased between July and October in all, except seven genotypes, and they were Sin 5–10 and Sac 4. In the mapping family, starch declined between July and October in all genotypes except Hyb 5 and 21. The parents of the mapping family are phylogenetically similar to Sac 4 (female parent) and Sin 5 (male), and so in showing a decline between the two time points the mapping family behaved like the two parental types but unlike the hybrids of the mixed population. The maximum NSC was 410 g plant^−1^ in Hyb 2 in October. The four hybrids of the mixed population produced the highest yields of NSC but after them was Hyb 13 of the mapping family in July (151 g plant^−1^) which was also the highest yielding plant of the mapping family. If harvests were to be shifted to capture maximum NSC (with no other considerations), the mixed population should be harvested in October, but the mapping family should be harvested in July.

To compare how yields of carbohydrate from Miscanthus compared with maize, the maximum yield of carbohydrate in t ha^−1^ was projected. The plants used in our study were spaced plants so it was not possible to calculate yields in t ha^−1^ from the values of individuals. However, in a study comparing 15 diverse genotypes harvested in autumn (September–October), a maximum yield of 19 t ha^−1^ was reported (Clifton‐Brown *et al*., 2001). This maximum value is in agreement with the spring yields of *M. x giganteus* in the United Kingdom being reported at ~14 t ha^−1^ which +40% for an autumn harvest equals 19.6 t ha^−1^. Yields in July were considered to be 30% of peak harvest mass (Fig. 4b), which equalled 5.7 t ha^−1^. The genotypes that produced the highest yields were the hybrids of the mixed population (Hyb 1–4); therefore, their average carbohydrate concentrations were used to calculate potential maximum yields. The maximum potential yield of total NSC in July was 0.56 t ha^−1^, nearly all of which (0.52 t) was in the form of soluble sugar (Table 3). In October, potential yields of total NSC were 1.3 t ha^−1^, 68% of which was soluble sugar and the other 32% was starch (Table 3).

**Table 3:** Predicted yields (t ha^−1^) of nonstructural carbohydrates (NSC) from high‐yielding hybrids

	Projected yields t ha^−1^		
	Soluble sugar	Starch	Total NSC
July	0.52	0.04	0.56
October	0.89	0.41	1.30

### Saccharification potential

The accessibility of the cell wall carbohydrates at the two time points was assessed by calculating the saccharification potential of cell wall‐derived glucose and xylose. The amount of total cell wall glucose and xylose yielded from acid hydrolysis generally increased between July and October in the mixed population and was significantly different between genotypes and date (*P* = 0.005 and *P* < 0.001, respectively, for glucose yields, and *P* = 0.011 and *P* < 0.001, respectively, for xylose yields; Table 4). No significant differences between genotypes in acid‐released glucose or xylose were detected for the mapping family (Table 3). In contrast to the acid‐released glucose and xylose (in the mixed population), the amount of enzymatically released glucose declined in both sets of plants (*P* < 0.001 for both mix population and mapping family), with a single exception (Hyb 16) between the two time points (Table 3). The amount of enzymatically released xylose generally increased in both sets of plants between July and October but the exception to this was Hyb 1, 2 and 3 in which yields of xylose slightly declined. These differences were significant between time points for both sets of plants (*P* < 0.001) but were not different between genotypes of the mapping family (*P* = 8.12 and *P* < .001 for mapping family and mixed population, respectively; Table 3). In July and October in the mapping family, the amount of carbohydrate released by the enzymes was greater than acid hydrolysis (Table 4). In July, the difference was within the degree of error but in October the difference between enzyme and acid hydrolysis was >20 mg day^−1^ DW. A possible explanation could be a greater degree of acetylation in October which would make the samples more resistant to acid hydrolysis (Chen *et al*., 2012). An esterase enzyme was included in the enzyme cocktail which would have helped hydrolyse ester bonds in enzyme‐treated samples (Pawar *et al*., 2013).

**Table 4:** Saccharification potential of glucose and xylose in a mixed population and mapping family in July and October. N = 3 ± SE. Statistics show two‐way anova with genotype and date as factors *P* = ≤ 0.05

Mixed Population	Acid digestible carbohydrate				Enzymatic carbohydrate release				% Digestibility			
	July	October	July	October	July	October	July	October	July	October	July	October
Genotype	Glucose		Xylose		Glucose		Xylose		Glucose		Xylose	
Sin 1	432.9 ± 4.1	429.2 ± 17.8	275.2 ± 10.5	290.4 ± 9.7	286.2 ± 8.7	273.0 ± 3.6	257.6 ± 8.4	308.5 ± 1.7	66.1 ± 1.5	64.1 ± 3.7	93.7 ± 1.2	106.6 ± 3.1
Sin 2	429.7 ± 11.8	427.6 ± 9.5	259.0 ± 2.9	299.9 ± 5.6	315.7 ± 6.0	291.8 ± 13.5	256.0 ± 6.6	306.5 ± 13.9	73.5 ± 0.6	68.5 ± 4.4	98.8 ± 1.5	102.1 ± 3.5
Sin 3	410.0 ± 16.9	450.9 ± 8.5	268.7 ± 11.2	308.6 ± 5.3	311.1 ± 2.4	267.5 ± 8.5	250.5 ± 13.1	303.6 ± 2.4	76.3 ± 3.4	59.3 ± 1.0	93.1 ± 1.0	98.4 ± 1.0
Sin 4	422.0 ± 11.8	399.6 ± 11.0	278.7 ± 4.3	295.7 ± 6.7	287.9 ± 0.2	265.2 ± 4.1	252.0 ± 4.6	296.1 ± 5.3	68.4 ± 1.9	66.5 ± 1.7	90.5 ± 2.5	100.2 ± 0.7
Sin 5	397.1 ± 15.6	404.5 ± 12.2	279.2 ± 5.0	291.1 ± 3.5	308.2 ± 6.0	294.7 ± 11.7	262.3 ± 12.2	285.1 ± 8.7	78.0 ± 3.7	72.8 ± 1.3	93.9 ± 3.8	98.0 ± 3.0
Sin 6	410.7 ± 4.5	432.8 ± 20.9	233.8 ± 3.0	291.7 ± 4.3	326.0 ± 3.8	284.5 ± 4.6	242.1 ± 5.6	280.2 ± 4.7	79.4 ± 0.5	66.4 ± 4.3	103.7 ± 3.5	96.2 ± 3.0
Sin 7	413.2 ± 2.8	427.6 ± 12.3	240.0 ± 8.1	302.8 ± 1.0	330.7 ± 11.5	306.9 ± 12.2	247.6 ± 7.8	291.8 ± 4.9	80.0 ± 2.8	71.8 ± 2.2	103.5 ± 4.5	96.4 ± 1.6
Sin 8	415.0 ± 11.1	418.1 ± 12.6	257.8 ± 6.4	296.9 ± 6.1	335.3 ± 11.9	298.8 ± 2.0	263.5 ± 4.3	313.1 ± 8.0	80.8 ± 1.2	71.7 ± 2.5	102.3 ± 0.9	105.7 ± 4.1
Sin 9	430.0 ± 9.1	426.2 ± 2.1	264.0 ± 4.3	307.3 ± 11.3	329.7 ± 3.8	293.2 ± 0.3	267.2 ± 5.3	309.4 ± 3.6	76.8 ± 2.3	68.8 ± 0.4	101.2 ± 0.9	101.1 ± 3.9
Sin 10	402.0 ± 12.6	432.3 ± 3.4	250.9 ± 2.1	298.6 ± 3.2	327.3 ± 10.3	286.4 ± 4.8	254.0 ± 1.6	316.8 ± 6.2	81.6 ± 3.0	66.3 ± 1.2	101.2 ± 0.2	106.1 ± 2.5
Hyb 1	450.6 ± 14.0	468.4 ± 13.4	284.3 ± 13.1	270.0 ± 9.3	317.3 ± 5.4	265.7 ± 4.0	269.7 ± 7.6	242.2 ± 23.6	70.5 ± 1.0	56.9 ± 2.1	95.4 ± 4.3	89.3 ± 7.0
Hyb 2	421.6 ± 7.8	472.6 ± 6.0	277.6 ± 5.4	272.8 ± 13.2	319.6 ± 4.5	268.3 ± 6.1	270.2 ± 5.2	245.0 ± 12.8	75.9 ± 1.5	56.8 ± 2.0	97.4 ± 2.1	90.3 ± 5.7
Hyb 3	419.5 ± 6.7	474.0 ± 14.6	255.7 ± 5.7	262.0 ± 5.3	316.6 ± 7.8	274.7 ± 8.1	250.2 ± 8.0	231.2 ± 4.2	75.5 ± 1.2	58.0 ± 1.5	97.8 ± 1.3	88.3 ± 1.8
Hyb 4	407.1 ± 6.1	472.5 ± 13.9	260.7 ± 2.8	277.9 ± 3.3	314.7 ± 4.4	292.7 ± 8.2	264.2 ± 0.9	266.9 ± 17.3	77.3 ± 1.0	62.2 ± 2.8	101.4 ± 0.8	95.9 ± 5.3
Sac 2	436.3 ± 14.8	456.4 ± 17.5	285.0 ± 3.6	279.3 ± 7.9	322.7 ± 10.1	274.8 ± 6.6	280.3 ± 4.8	285.4 ± 7.4	74.3 ± 3.8	60.4 ± 2.1	98.5 ± 2.9	102.2 ± 1.7
Sac 3	404.5 ± 1.5	448.8 ± 2.3	262.9 ± 2.9	307.2 ± 5.1	325.0 ± 20.3	285.5 ± 10.9	276.4 ± 22.5	309.6 ± 3.7	80.3 ± 5.0	63.6 ± 2.3	104.9 ± 7.4	100.9 ± 1.9
Sac 4	416.2 ± 8.0	452.3 ± 2.4	254.7 ± 5.1	317.5 ± 3.6	322.0 ± 6.5	271.4 ± 5.4	265.5 ± 9.0	307.5 ± 6.2	77.5 ± 2.7	60.0 ± 1.3	104.2 ± 2.9	96.8 ± 1.9
Sac 5	412.1 ± 7.7	483.3 ± 10.8	284.1 ± 5.4	287.9 ± 8.3	293.5 ± 13.7	266.7 ± 6.4	261.5 ± 4.7	277.5 ± 14.2	71.4 ± 3.9	55.4 ± 2.5	92.2 ± 3.2	96.3 ± 3.0
Average	418.4 ± 3.2	443.2 ± 5.9	265.1 ± 3.6	292.1 ± 3.5	316.1 ± 3.4	281.2 ± 3.1	260.6 ± 2.4	287.6 ± 6.2	75.8 ± 1.0	63.9 ± 1.3	98.5 ± 1.1	98.4 ± 1.3
Genotype	0.005		0.011		0.001		<0.001		<0.001		0.055	
Date	<0.001		<0.001		<0.001		<0.001		<0.001		0.902	
Geno × Date	0.038		<0.001		0.703		<0.001		0.07		0.151	

Mapping family	Acid digestible carbohydrate				Enzymatic carbohydrate release				% Digestibility			
	July	October	July	October	July	October	July	October	July	October	July	October
Genotype	Glucose		Xylose		Glucose		Xylose		Glucose		Xylose	
Goliath	423.2 ± 6.8	404.9 ± 0.5	255.5 ± 9.0	271.0 ± 3.2	305.9 ± 6.1	257.6 ± 7.5	253.0 ± 12.6	300.5 ± 12.4	72.3 ± 1.3	63.6 ± 1.9	98.9 ± 1.5	111.1 ± 5.9
Hyb 5	465.4 ± 16.8	441.0 ± 3.5	258.6 ± 24.0	287.9 ± 9.6	318.5 ± 39.7	311.5 ± 9.7	264.0 ± 28.9	324.9 ± 2.9	68.4 ± 6.0	70.9 ± 2.6	102.0 ± 26.4	113.1 ± 4.9
Hyb 6	430.4 ± 2.6	419.0 ± 4.7	255.2 ± 10.7	288.4 ± 16.5	299.6 ± 15.5	273.9 ± 5.0	258.1 ± 23.7	308.3 ± 5.9	69.8 ± 3.2	65.4 ± 0.4	103.9 ± 11.3	107.2 ± 6.4
Hyb 7	416.8 ± 7.6	445.2 ± 4.0	286.8 ± 22.6	278.1 ± 3.0	353.8 ± 10.3	316.6 ± 9.1	289.3 ± 1.6	326.7 ± 6.0	85.0 ± 1.6	71.1 ± 1.8	100.8 ± 9.4	117.5 ± 2.8
Hyb 8	451.9 ± 13.1	405.9 ± 5.8	243.0 ± 10.3	277.0 ± 11.4	364.8 ± 6.3	320.9 ± 6.3	261.7 ± 5.9	305.5 ± 6.9	80.8 ± 1.5	79.5 ± 0.6	107.6 ± 3.8	110.5 ± 2.0
Hyb 9	417.3 ± 2.8	440.3 ± 6.5	272.3 ± 7.4	293.5 ± 2.2	299.8 ± 13.9	284.8 ± 18.3	257.8 ± 10.7	315.4 ± 13.6	71.9 ± 2.7	65.5 ± 4.0	94.6 ± 3.2	107.4 ± 4.2
Hyb 10	452.6 ± 7.2	438.0 ± 11.0	258.7 ± 9.3	297.0 ± 5.7	317.4 ± 2.3	265.8 ± 3.4	269.3 ± 9.1	310.2 ± 3.7	70.1 ± 1.5	60.7 ± 2.5	106.6 ± 1.8	104.5 ± 0.8
Hyb 11	457.2 ± 15.1	436.9 ± 5.8	265.4 ± 11.7	280.2 ± 6.0	322.0 ± 3.8	273.8 ± 10.2	263.7 ± 11.3	303.4 ± 23.0	70.5 ± 2.5	62.6 ± 2.3	99.8 ± 2.3	108.6 ± 7.9
Hyb 12	466.4 ± 10.4	431.7 ± 6.6	267.1 ± 5.7	284.6 ± 2.1	350.4 ± 7.2	310.7 ± 5.9	277.9 ± 8.5	321.2 ± 15.1	75.1 ± 3.4	72.0 ± 1.3	104.1 ± 1.3	112.8 ± 6.4
Hyb 13	463.7 ± 28.3	425.4 ± 22.3	279.2 ± 4.9	292.4 ± 5.1	329.2 ± 26.6	276.7 ± 5.0	274.7 ± 8.9	306.3 ± 12.9	71.1 ± 2.6	65.2 ± 2.5	98.4 ± 3.4	104.8 ± 2.8
Hyb 14	428.8 ± 5.8	422.1 ± 9.8	226.1 ± 15.4	288.5 ± 5.9	334.1 ± 7.4	285.7 ± 3.3	263.9 ± 16.5	298.6 ± 9.4	78.2 ± 1.6	67.7 ± 2.4	116.9 ± 5.2	103.6 ± 4.8
Hyb 15	450.0 ± 6.5	425.8 ± 4.4	274.5 ± 4.4	264.5 ± 4.1	349.5 ± 3.3	314.2 ± 6.8	279.0 ± 4.7	290.7 ± 11.3	77.9 ± 1.1	73.8 ± 1.1	101.6 ± 1.1	110.0 ± 3.6
Hyb 16	461.5 ± 10.7	436.7 ± 12.4	259.5 ± 8.1	275.8 ± 6.6	339.4 ± 2.5	355.8 ± 6.4	273.5 ± 6.3	318.7 ± 15.8	70.9 ± 2.3	81.5 ± 2.1	64.3 ± 1.3	116.1 ± 4.6
Hyb 17	428.7 ± 6.5	417.5 ± 5.2	275.4 ± 13.5	298.3 ± 4.1	290.8 ± 21.3	279.2 ± 6.4	271.5 ± 18.6	298.1 ± 7.9	67.5 ± 3.9	67.3 ± 1.5	98.6 ± 7.2	99.8 ± 4.1
Hyb 18	483.9 ± 4.8	406.2 ± 8.3	273.9 ± 4.0	275.7 ± 4.2	382.8 ± 5.0	296.7 ± 3.2	277.5 ± 4.9	301.4 ± 6.8	79.1 ± 1.6	73.2 ± 2.1	101.6 ± 1.6	109.5 ± 3.6
Hyb 19	460.1 ± 3.4	423.8 ± 15.0	254.2 ± 6.3	285.1 ± 15.0	322.3 ± 8.2	267.3 ± 14.4	264.3 ± 9.3	306.2 ± 13.2	70.1 ± 1.7	63.0 ± 4.1	104.0 ± 1.4	107.4 ± 2.2
Hyb 20	456.4 ± 20.4	407.4 ± 8.6	274.1 ± 20.5	266.0 ± 5.3	320.1 ± 11.2	282.0 ± 3.7	283.9 ± 12.4	297.0 ± 11.4	70.3 ± 2.5	69.4 ± 1.3	103.7 ± 12.0	111.6 ± 5.8
Hyb 21	463.5 ± 10.8	437.7 ± 7.0	235.4 ± 4.8	296.9 ± 3.5	365.0 ± 3.8	329.2 ± 8.6	241.9 ± 7.7	294.1 ± 4.2	78.7 ± 1.3	75.2 ± 1.7	103.3 ± 1.2	100.1 ± 1.7
Hyb 22	473.7 ± 8.9	435.7 ± 11.4	253.8 ± 7.1	280.4 ± 11.8	396.9 ± 3.7	297.5 ± 14.4	256.6 ± 8.9	312.8 ± 10.7	83.7 ± 1.3	68.3 ± 5.6	101.4 ± 0.7	111.7 ± 2.3
Hyb 23	471.2 ± 6.5	407.1 ± 23.9	254.7 ± 2.9	272.5 ± 2.5	320.9 ± 1.9	260.0 ± 15.8	264.6 ± 9.4	289.6 ± 12.7	68.1 ± 1.4	64.0 ± 6.0	103.9 ± 2.5	106.4 ± 3.5
Average	451.1 ± 4.5	425.4 ± 3.0	261.2 ± 3.4	282.7 ± 2.3	334.2 ± 6.4	293.0 ± 5.8	267.3 ± 2.5	306.5 ± 2.4	74.0 ± 1.2	69.0 ± 1.3	100.8 ± 2.2	108.7 ± 1.1
Genotype	0.105		0.476		<0.001		0.812		<0.001		0.963	
Date	<0.001		<0.001		<0.001		<0.001		<0.001		0.002	
Geno × Date	0.052		0.12		0.1		0.973		0.239		0.767	

The % digestibility of cell wall glucose significantly declined between the two time points for both sets of plants whereas the % digestibility of xylose showed a nonsignificant change between July and October. The average difference in % digestibility of glucose was −16% of July levels in October for the mixed population and −7% of July levels in October for the mapping family. However, as biomass increased by 70% between July and October, yields of digestible sugars in October will still greatly exceed yields in July.

### Nutrient remobilization

**Figure 7:**
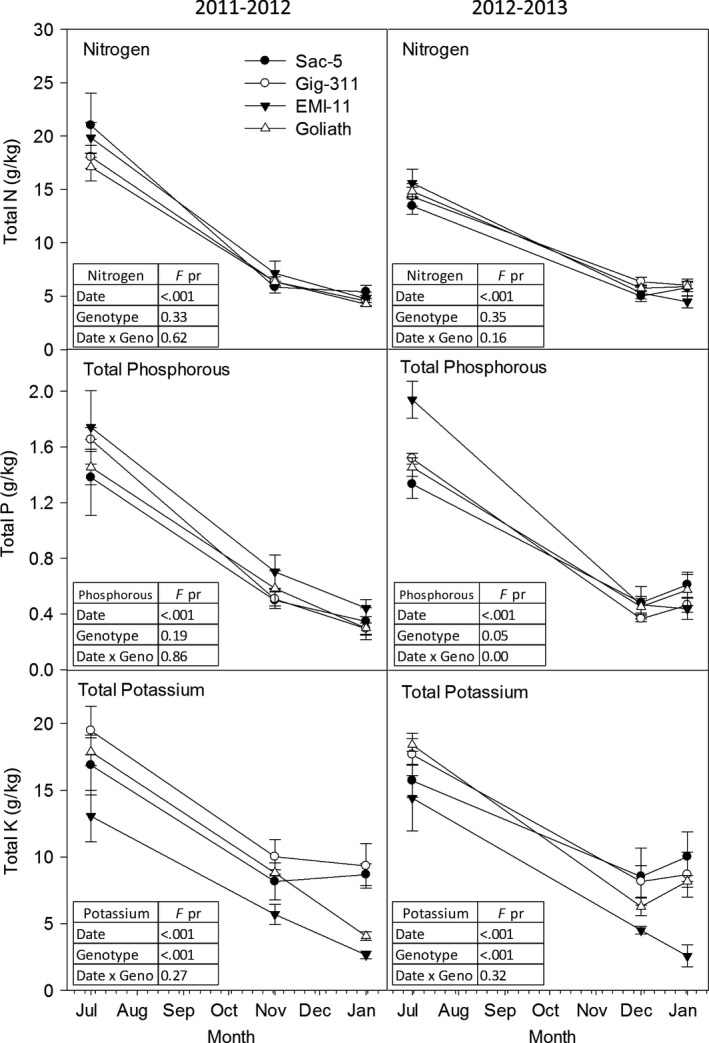
Total N, P and K in above‐ground material (leaf and stem) in summer, autumn and winter for 2 years. N = 4 ± SE. Statistical analyses show results of anova (*P* = ≤ 0.05).

The nitrogen (N), phosphorous (P) and potassium (K) of the total above‐ground material was analysed at six time points over 2 years: July (2011 & 2012), November (2011), December (2012) and January (2011 & 2012; Fig. 7). The climate data are shown in Fig. 1. Significant differences were observed between the harvest dates for all nutrients in both years (*P* =< 0.01; Fig. 7). In July 2011 and 2012, N concentration was 13–21 g kg^−1^ but by January this had declined three‐ to fourfold to be only 5 g kg^−1^. A similar fourfold decline was also seen in P over both years. No significant differences were observed between genotypes for N and significant differences between genotypes were only observed for P in 2012 (*P* = 0.05; Fig. 7). However, the decline of K was different between genotypes. In both years, the abundance of K in Sac 5 and Gig 311 declined by ~50% between July and January whereas EMI‐11 declined by ~80% and Goliath declined by 77% in 2011–2012 and 55% in 2012–2013 (Fig. 7). Potassium was the only nutrient to show a significant difference between genotypes for both years (*P* =< 0.01). With the exception of P in 2012, no interactions between date and genotype were detected for any element in either year. In 2011, EMI‐11 and Goliath showed a clear downward trend for K from November to January but this was not observed in Sac‐5 or Gig‐311. In 2011, the maximum change in N and P from November to January was <0.3 g kg for all genotypes whereas K in EMI‐11 and Goliath declined by an average of 3 and 5 g kg, respectively. In 2012, no changes in nutrient concentrations were observed between December and January with the exception of EMI‐11 (very slightly) for N and more clearly for K.

These results suggest that for Sac‐5 and Gig‐311, moving harvest date back to autumn would not leave the rhizome depleted of nutrients. However, an earlier harvest may have an impact on the *M. sinensis* genotypes, particularly levels of K in EMI‐11. If stems were harvested in July, the addition of approximately 18 g kg^−1^ of N, 1.6 g kg^−1^ of P and 13–20 g kg^−1^ of K would be required to replenish the rhizome.

## Discussion

### NSC abundance

Two of the genotypes used in this study, Sac 5 and Goliath, are the same as those used in previously published studies of carbohydrate dynamics (Purdy *et al*., 2013, 2014, 2015). Previously published reports from 2011 and 2012 of Sac 5 and Goliath found concentrations of carbohydrate to be 7% and 6% NSC in July and 3% and 5% NSC in November, respectively. These are slightly lower than the findings of this paper where Goliath had 10% NSC and Sac 5 had 9% in July and Goliath had 3% and Sac 5 had 8% in October. The summer values are higher in our current study by about 30% for both genotypes, and the autumn level was 40% higher in Sac 5 (Goliath was the same in both studies in autumn). The difference in the concentrations was probably caused by the warmer, drier summer in 2013 compared to 2011 and 2012. It has previously been shown that PAR and maximum temperature showed the strongest correlation with NSC abundance in four genotypes of *Miscanthus* (Purdy *et al*., 2014). Furthermore, in this aforementioned study, Goliath and Sac 5 were grown in replicated plots at two sites in the United Kingdom; one in west Wales and the other in south‐east England. The English site had higher PAR and daily temperatures than the Welsh site (Cunniff *et al*., 2015), and soluble sugars were on average 40% higher in both Goliath and Sac 5 in July at the English site compared to w. Wales (Purdy *et al*., 2014). Therefore, the hotter drier summer probably resulted in the higher concentrations of carbohydrates observed in this study compared to those of Purdy *et al*. (2014).

The timing of an autumn harvest is dependent on climate at the growing site as this will determine the rate of carbohydrate remobilization from the stems to the rhizome during senescence. In a comparison between west Wales and south‐east England, all genotypes in Wales had retained carbohydrates in the stems at the end of winter, whereas in s.e England nearly all carbohydrate had been remobilized by November (Purdy *et al*., 2014). This response was found to be correlated with the minimum daily temperature which is lower in s.e England than in w. Wales, which receives warmer air from the Gulf Stream. Therefore, the potential yields of carbohydrate in autumn will depend on the local climate, and harvest date will have to be optimized for different regions.

According to the recommended maize variety list released by the National Institute of Agricultural Botany (NIAB), the t ha^−1^ of starch in forage maize varieties averaged 6 t ha^−1^ on favourable sites (NIAB, 2016) which is five times greater than the highest projected yields of *Miscanthus* in our study. However, the economic and environmental cost of growing forage maize is greater than *Miscanthus*. Economically, it costs £411 ha^−1^ to grow and harvest maize whereas it costs £231 ha^−1^ to grow *Miscanthus* (Nix, 2016). Therefore, it is financially cheaper to grow *Miscanthus*. Environmentally, there is great concern about maize cropping because it is associated with soil erosion, agrochemical leakages into waterways and low biodiversity (EEA, 2006, Palmer & Smith, 2013). In a study of the biomethane potential (BMP) of a number of potential alternatives to maize, *Miscanthus* silage harvested in autumn was identified as the most competitive candidate (Mayer *et al*., 2014b). In Germany, the increased yields of *Miscanthus* (26 t ha^−1^ in October) compared to the United Kingdom already make it competitive against maize for biogas production (Kiesel & Lewandowski, 2016). *Miscanthus* sequesters carbon in the soil, and N_2_O emissions can be five times lower under unfertilized *Miscanthus* compared to annual crops (Clifton‐Brown *et al*., 2007; McCalmont *et al*., 2015). Therefore, *Miscanthus* also ‘wins’ in terms of its environmental impacts. However, the fact that cannot be denied is that the yields of NSC which are positively correlated with BMP (Whittaker *et al*., 2016) are currently at least five times lower in *Miscanthus*.

### Breeding potential

*Miscanthus* has undergone no selective breeding for NSC composition unlike other grass crops such as sugarcane and high sugar *Lolium Perenne* (ryegrass). For example, in *Lolium*, selective breeding led to a 31% increase in water‐soluble carbohydrates between 1994 and 2000 (Wilkins & Lovatt, 2011). The idea of breeding *Miscanthus* as a temperate sugarcane has been previously suggested (de Souza *et al*., 2013), but in this study we have shown that the highest concentration of NSC found in any of our genotypes was starch which reached concentrations of up to 20% DW in summer in the mapping family. Sugarcane is exceptional amongst grasses for storing its sugars in the central vacuoles of the internode parenchyma, which is presumably an adaptation that *Miscanthus* does not possess (Glasziou & Gayler, 1972). The accumulation of high levels of soluble sugars has been shown to repress photosynthesis in a number of species including sugarcane. This has been suggested to be the reason that yields of sugar in sugarcane have only increased through increases in biomass, not sugar concentrations, for several decades (Jackson, 2005; McCormick *et al*., 2008). In contrast, starch presents an inert form of stored glucose that accumulates in the chloroplast (Zeeman *et al*., 2007). In transgenic maize engineered to accumulate high levels of leaf starch through RNAi of the *GLUCAN WATER DIKINASE* gene, starch was increased 20‐fold in the transgenic plants with no impact on total biomass (Weise *et al*., 2012). Therefore, rather than targeting soluble sugars for improvement, it may be more logical to target starch as a final product, avoiding repression of photosynthesis. As has been previously observed, there appeared to be a negative relationship between starch and yield (Purdy *et al*., 2015); for example, Hybs 21 and 22 had the highest concentration of starch in July but were amongst the lowest yielding plants. However, there were exceptions to this trend; Hyb 5 was classified as a medium‐yielding plant and still contained 14% starch in summer. Furthermore, in five plants of the mixed population, starch levels in autumn were higher than in summer indicating that this form of carbohydrate can be accumulated later into the year. For example, if the later carbohydrate accumulating habit of the high‐yielding genotype Hyb 4 could be combined with the exceptionally high levels found in medium‐yielding Hyb 5, heterosis in the progeny could lead to high levels of starch accumulation in autumn. The higher levels of lignin in *Miscanthus* require a longer retention time in digesters, and so targeting lignin reduction through breeding would also increase digestibility (Kiesel & Lewandowski, 2016). The cutting tolerance of *Miscanthus* is dependent upon its rhizome carbohydrate reserves (Kiesel & Lewandowski, 2016) which could contradict our suggestion that starch should be targeted for accumulation and removal during harvest. However, it has previously been shown that the high‐yielding *M. x giganteus* (Gig‐311) and average yielding Sac 5 both retain carbohydrates in their stems in autumn and winter even after frosts which would have killed the stems and with them the starch digesting enzymes required for conversion of starch to sucrose for transport to the rhizome (Purdy *et al*., 2014). Therefore, it appears that at least some genotypes can tolerate the removal of starch without detriment to their ongoing sustainability, probably owing to refilling earlier in the year (Purdy *et al*., 2014) which is a characteristic that should be retained in new varieties (Kiesel & Lewandowski, 2016).

### Saccharification potential and nutrient remobilization

As *Miscanthus* matures, changes occur within the cell wall including the increased accumulation of cell wall and ester‐linked phenolic acids and lignin (Ngoc Huyen *et al*., 2010; da Costa *et al*., 2014). The concentration of lignin, its composition and the manner in which it binds holocellulose within the cell wall are often seen as exacerbating factors of cell wall recalcitrance to enzymatic deconstruction (da Costa *et al*., 2014). In agreement with previous reports, the % digestibility of cellulose declined between July and October. Therefore, if the crop consisted of the mapping family (or a member thereof), it would make most sense to harvest in July because the total sugar yields and saccharification potential are higher but analysis of the nutrient data suggests that the impact of this decision could be considerable. In contrast, in Gig‐311 and Sac 5, no differences were observed in the concentrations of N, P or K between November and December and January when the crop is usually harvested for final biomass. The date of the first frost in 2011 and 2012 was 07 November 2011 and 28 November 2012, respectively (Purdy *et al*., 2014). The plants were harvested on 14 November 2011 and 04 December 2012. Therefore, in 2011, the plants were harvested after the first frost which would have killed the above‐ground stems so any remaining carbohydrates or nutrients could not have been remobilized to the rhizome. The closeness of the point between our harvests and the first frosts probably accounts for the lack of change in N, P and K between autumn and early spring (January).

Despite the decrease in saccharification potential in autumn, the additional biomass yield compensates for this decline. For example, the mean yield of enzyme‐digestible glucose from cellulose for both sets of plants in July and October was approximately 32% and 29% DW, respectively. If average July and October yields of 4 and 12 t ha^−1^ are assumed, this equates to cellulose yields of 1 and 3 t ha^−1^, in July and October, respectively. Moreover, several studies have shown no detrimental effect of autumn harvest on yield although the duration of years that this practice can be maintained is unclear. In Germany, no negative effect on yield was observed following 3 years of autumn harvests but in France yields were maintained for 4 years with no additional fertilization but then suddenly dropped in the fifth year and required the addition of N to return to the previous tonnage (Mayer *et al*., 2014b; Yates *et al*., 2015). In a recent study, yields of M. × giganteus in the year proceeding an October harvest were actually slightly higher than when plants had been harvested in winter (Kiesel & Lewandowski, 2016). Therefore, it is likely that, as with carbohydrates, the rate and timing of N P K remobilization may well vary with site and climate and the sustainability of shifting the harvest date forward would have to be assessed at a wider range of locations. In agreement with previous reports (Cadoux *et al*., 2012), the recycling of K was less efficient and showed greatest variation between genotypes. It is highly likely that this results in different genotypic demands for replenishment of K, regardless of whether the harvest is in autumn or early spring, with genotype such as EMI‐11 requiring less replenishment than a genotype such as Gig‐311. A major difference between EMI‐11 and Gig‐311 is flowering; EMI‐11 flowers early whereas Gig‐311 rarely flowers at Aberystwyth, and if it does, it occurs in autumn (Purdy *et al*., 2014). Therefore, effective remobilization of nutrients may be linked to the completion of the annual life cycle (of a perennial) through flowering.
